# Investigating the Role of State Permitting and Agriculture Agencies in Addressing Public Health Concerns Related to Industrial Food Animal Production

**DOI:** 10.1371/journal.pone.0089870

**Published:** 2014-02-24

**Authors:** Jillian P. Fry, Linnea I. Laestadius, Clare Grechis, Keeve E. Nachman, Roni A. Neff

**Affiliations:** 1 Center for a Livable Future, Johns Hopkins University, Baltimore, Maryland, United States of America; 2 Department of Environmental Health Sciences, Johns Hopkins Bloomberg School of Public Health, Baltimore, Maryland, United States of America; 3 Joseph J. Zilber School of Public Health, University of Wisconsin-Milwaukee, Milwaukee, Wisconsin, United States of America; 4 Department of Health Policy and Management, Johns Hopkins Bloomberg School of Public Health, Baltimore, Maryland, United States of America; Kagoshima University Graduate School of Medical and Dental Sciences, Japan

## Abstract

**Objectives:**

Industrial food animal production (IFAP) operations adversely impact environmental public health through air, water, and soil contamination. We sought to determine how state permitting and agriculture agencies respond to these public health concerns.

**Methods:**

We conducted semi-structured qualitative interviews with staff at 12 state agencies in seven states, which were chosen based on high numbers or rapid increase of IFAP operations. The interviews served to gather information regarding agency involvement in regulating IFAP operations, the frequency and type of contacts received about public health concerns, how the agency responds to such contacts, and barriers to additional involvement.

**Results:**

Permitting and agriculture agencies’ responses to health-based IFAP concerns are constrained by significant barriers including narrow regulations, a lack of public health expertise within the agencies, and limited resources.

**Conclusions:**

State agencies with jurisdiction over IFAP operations are unable to adequately address relevant public health concerns due to multiple factors. Combining these results with previously published findings on barriers facing local and state health departments in the same states reveals significant gaps between these agencies regarding public health and IFAP. There is a clear need for regulations to protect public health and for public health professionals to provide complementary expertise to agencies responsible for regulating IFAP operations.

## Introduction

**Figure 1:**
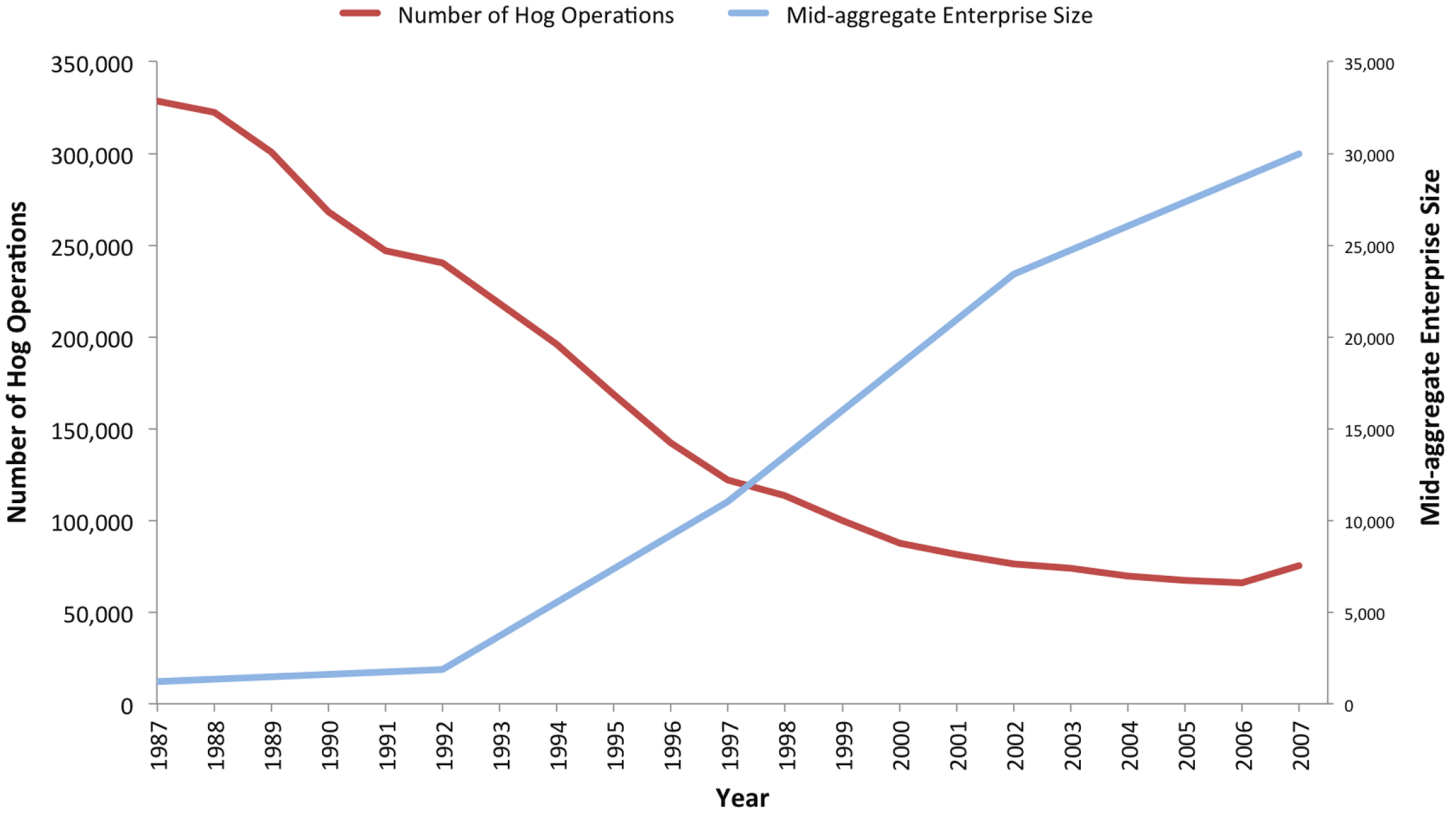
Hog production in the US: Number of operations and mid-aggregate enterprise size; 1987–2007. Data from the United States Department of Agriculture (USDA) shows the decline in number of hog operations and growth in operation size from 1987 to 2007. Sources: The Changing Organization of U.S. Farming, USDA Economic Research Service (http://www.ers.usda.gov/amber-waves/2011-december/changing-farming-practices.aspx#.UgqVWOu9xEo) and USDA Census of Agriculture QuickStats, various years (http://quickstats.nass.usda.gov/?source_desc=CENSUS).

A dramatic series of changes in the landscape of animal agriculture have taken place over the past seventy years, accompanied by multiple public health concerns [1]. Small farms raising a diversity of crops and food animals have increasingly and steadily given way to a model of industrial food animal production (IFAP) that raises large numbers of animals in concentrated quarters, often on farms whose only crops are animal feed grown on spray fields. Hog production, for example, has shifted significantly since the 1980s. As Figure 1 illustrates, from 1987 to 2007 the number of hog operations in the US decreased from over 320,000 to about 75,000 [2]. Mid-aggregate enterprise size is a measurement of farm production that shows the point where half of a product comes from larger farms, and half from smaller farms. In 1987, the mid-aggregate enterprise size of US hog farms was 1,200 hogs, according to the USDA. By 2007, this measurement increased to 30,000, reflecting a 2,400% increase [3], as shown in Figure 1. The mid-aggregate enterprise size of dairy and broiler (chicken meat) production operations increased by 613% and 127%, respectively, during the same interval [3]). There has also been geographic concentration in production. In 2007, 75% of all U.S. hogs were raised in only 220 counties, down from 508 counties in 1987 [3]. These changes in the model, methods, and system within which animals are produced for food pose both a regulatory challenge and clear concerns for environmental health and public health more generally.

Research linking IFAP to public health concerns and impacts continues to increase. In addition to posing respiratory health risks to those residing near operations [4]–[8] due to emissions that include hydrogen sulfide [9], particulate matter [9], endotoxins [10], ammonia [11], allergens [12], and volatile organic compounds [13], [14], odor generated by IFAP operations and spray fields has been associated with a broad range of health problems. Public access to information regarding hazardous airborne releases from IFAP operations is hindered due to exemptions in federal laws that require disclosure of such releases [15], despite research linking chronic exposure to odors from IFAP to headaches, nausea, upset stomach, mood disorders, high blood pressure, and sleep problems [16]–[20]. Additionally, there is growing evidence that livestock can transmit methicillin-resistant *Staphylococcus aureus* (MRSA) to humans [21]–[23]. Rural water supplies are also at risk, as IFAP-generated animal waste contaminants, including nitrates, pathogens, pharmaceuticals, metals, and hormones, can leach into ground water [24], [25]. All of these concerns may be compounded by the fact that IFAP operations are disproportionately located in low-income communities with high-percentages of minority populations [26]–[28], which are more likely to experience limited political power [29] and barriers to healthcare access [30].

Despite health risks posed by these operations, regulation of IFAP is limited and characterized by a patchwork of different regulatory approaches from state to state. Under the Clean Water Act (CWA), IFAP operations that are designated as Concentrated Animal Feeding Operations (CAFOs) are required to obtain National Pollution Discharge Elimination System (NPDES) permits in order to discharge into U.S. waterways. Designation as a CAFO is determined by size and potential to pollute the surrounding environment. States are granted authority to determine some requirements, issue NPDES permits, monitor compliance, and impose penalties by the U.S. Environmental Protection Agency if the state adopts federal requirements into law. As of 2008, 44 states had permitting authority for CAFOs [31]. Animal operations that fail to meet the technical definition of a CAFO may still cause numerous concerns, thus the more inclusive term IFAP is utilized here.

The state agency responsible for permitting IFAP operations varies among states, with almost all delegating the responsibility to Departments of Environmental Protection/Management, Natural Resources, or Agriculture [31]. The organization and jurisdiction of state agencies responsible for regulations pertaining to the environment and natural resources varies significantly by state [32]; so defining the broader roles and responsibilities of these agencies is difficult. We focused on environmental agencies that have been given authority by the EPA to implement the NPDES permitting program for CAFOs, and we refer to these agencies as “permitting agencies”. Responsibilities of Departments of Agriculture vary less by state, and in general they include promotion and regulation of agriculture in the state, as well as other common responsibilities such as conservation and farmland protection [33]. Regulatory authority is shared among agencies in some states, and the transfer of regulatory authority to Departments of Agriculture has been favored by industry [34]. Some states have developed additional regulations for smaller IFAP operations that do not meet the size threshold to be considered a CAFO. Additionally, states vary regarding resources available to monitor and enforce regulations, and potential penalties for violations [31], [35]. Common across most states, however, is delegating the permitting to an agency without a primary mandate to address public health [31], raising concerns that public health issues may not be adequately monitored or addressed by the agencies tasked with regulating IFAP operations.

A 2008 report by the National Conference on State Legislatures provided an overview of agencies in each state responsible for regulating IFAP [31], but the report did not address how state agencies respond to community health concerns arising from IFAP operations. We conducted interviews with state permitting agency and agriculture department staff members to determine the ability of these agencies to: 1) monitor IFAP operations and enforce current regulations, and 2) respond to citizen health concerns arising from IFAP operations. This manuscript represents the second part of a study examining how state and local agencies address health concerns associated with IFAP. Our previously published research [36] found that despite the evidence linking IFAP to public health concerns, state and local health departments play a limited role in addressing health issues linked to IFAP operations. Health departments report a lack of formal jurisdiction over IFAP operations as the primary barrier to regulatory response. As a result, we found that many health departments refer IFAP concerns, including community member concerns related to health, to departments of agriculture and permitting agencies.

Assessing the roles of the main agencies with jurisdiction over issues relevant to public health is essential to a full understanding of how health concerns, including those related to IFAP, are addressed. In fact, defining inter-organizational relationships, evaluating capacity as financial priorities shift, and identifying gaps in services or jurisdiction are key priorities in public health systems research [37], [38]. We aimed not only to characterize the current role, capacity, and barriers of the primary agencies potentially involved with IFAP and public health, but also to highlight gaps in agency responsibilities that would not be identified if only one type of agency were studied.

## Methods

Given the absence of research on the role of state agriculture and permitting agencies in responding to health concerns stemming from IFAP, an exploratory qualitative approach was chosen for data collection and analysis. See Fry (2013) for further details on the initial study, which examined state and county health department responses [36]. An inductive qualitative research approach was used, with a focus on flexibility rather than allegiance to any specific theoretical approach [39], [40]. Important aspects of study design, data collection, and data analysis are described below.

We used a purposive sampling strategy to select the states included in this study. First, all U.S. counties were ranked by USDA Census of Agriculture hog inventory data in two ways: 1) 2007 county hog inventory of operations with 1,000 or more hogs, and 2) increase in hog inventory of 1,000+ head operations between 2002 and 2007. It was anticipated that many hogs produced in intensive settings or large increases in hog numbers could lead community members or others to request that agencies take action to address health concerns. We also sought to examine settings where hog operations were near residential areas by ranking the top sixty counties from each list by population density using 2000 Census data. We then selected the top fifteen counties by population density from the two lists. These counties were located in eight states. Although states were chosen based on hog production and census data, the interviews asked about IFAP in general, and did not focus solely on hog operations.

We contacted state agriculture and permitting agency employees in the eight states to perform semi-structured interviews. Both types of agencies were included in order to understand their roles and to identify potential gaps in agency responses to health concerns. Permitting agencies and some agriculture departments were identified through the National Conference on State Legislatures’ Survey of State Policies on CAFOs [31]. Through web-based searching and investigation, we confirmed the information from the report and collected contact information for additional agriculture departments. Most commonly, permitting responsibilities fell under the authority of departments of environmental protection or natural resources. We contacted staff members who worked on livestock permitting and/or livestock production, identified using agency websites. The specific roles and titles of interviewees varied across states and included, among others, livestock permitting and water quality specialists, state veterinarians, and community-relations liaisons. In some cases, staff we contacted referred us to others in their agencies who could better answer our questions.

All interviews were conducted over the telephone by JF or LL, with a note-taker present on each call to document responses. Interviews were not audio recorded. The questionnaires included mostly open-ended and some closed-ended questions (See Appendix S1 for survey instruments), and some follow-up or clarifying questions were asked based on participants’ responses. We also specifically queried permitting agency staff members about their states’ permitting regulations. Interview questions were developed based on our prior familiarity with the topic, which is drawn from extensive background reading, original research, and interactions with community members impacted by IFAP operations. We did not use the terms, IFAP or CAFO, in the interviews. Before beginning each interview, we read participants a confidentiality statement. The Johns Hopkins Bloomberg School of Public Health Institutional Review Board (IRB) determined the study was exempt from IRB oversight and did not require an informed consent process.

After data collection was complete, notes from three interviews were double coded through an inductive coding process using *HyperRESEARCH 3.0.3* (ResearchWare, Randolph, MA). Codes were jointly discussed to develop a uniform codebook, which was then applied to the remaining interviews. As this is a qualitative study with a small, purposive sample, we provide limited numeric information to avoid implying that the findings are generalizable to a larger population [41]. Instead, we describe important themes identified in the interviews that allow better understanding of the situation in states with significant industrial hog production.

## Results

We conducted telephone interviews with staff members from permitting agencies in seven states and departments of agriculture in five states. In one state, both the permitting agency and department of agriculture declined to participate, and agriculture department staff in two other states also declined. In total, twelve interviews in seven states were conducted between November 2010 and October 2011. Most of the interviews lasted between 35 and 45 minutes.

### Regulatory Oversight

In nearly all instances, state-level environment or natural resource departments managed NPDES permits. One state delegated permitting authority to counties. In two states, departments of agriculture shared regulatory authority with other departments, and in another state the permitting agency held only narrow authority over NPDES permitting with many activities transferred to the agriculture department in recent years.

As specified in the federal Clean Water Act, NPDES permitting requirements apply only to certain IFAP operations that match the defined criteria for CAFOs. States also reported additional non-NPDES regulatory measures. For example, some states require permits for IFAP operations that do not match the federal CAFO definition, although requiring less information for approval than for CAFOs. Some permitting agency staff members also said they were trying to develop regulations that would strengthen reporting requirements for small/medium size operations, thereby bringing them in line with CAFO permitting requirements. One agriculture department staff member noted that, “Issues are more frequently from smaller farms, but people are quick to blame bigger farms”.

We asked interviewees if their agencies had any setback/zoning policies related to human health concerns. While several counties and states require that IFAP operations be a certain distance away from property lines, wells, waterways, homes, churches, schools, and/or parks, many interviewees said the requirements are not necessarily based on health standards. One staff member said,

“There are setbacks, but those are more for odor rather than health. This can be from residences or populated areas, it’s just a form of odor management. There are some setbacks for wells/private water wells too, that have more direct relevance to public health issues.”

Setbacks can reduce public health impacts of odor and poor air quality, but interviewees indicated that most setbacks are not based on evidence-based health standards specifically designed to limit exposure to gases and particulate matter emitted from IFAP operations.

One permitting agency staff member said two counties in the state had established more stringent requirements for setbacks through health ordinances. Two states reported having air quality standards that apply to IFAP operations, but in one state the standard only applies to new operations.

### Inspections

States varied significantly in the frequency of inspections of permitted CAFOs for compliance with regulations. One permitting agency staff member said that they “can only afford to [inspect] on a complaint basis” because they “don’t have staff or money.” By contrast, a staff member from another state’s agriculture department indicated that they inspected CAFOs every six months. The staff member also stated, “before reforms a few years ago, some facilities had gone 25 years without inspection.”

### Contacts and Concerns Reported

All interviewees noted that their agencies had been contacted by people concerned about issues associated with living or spending time near animal production operations and/or manure (i.e., manure storage or spray fields). Estimates from permitting agency staff ranged from 25 to 120 contacts per year, and agriculture agency staff estimates ranged from fewer than 5 times per year to an average of 5 times per week. Some interviewees said that calls were generally more frequent when new or expanding operations were proposed and that they believed that calls had decreased due to the implementation of additional regulations. The absence of regulations pertaining to common community concerns with IFAP also appeared to reduce contacts as people began to learn what agencies were able to do in response to concerns. One permitting agency staff member said there “used to be more calls about odor, but there are no odor regulations, so there is nothing we can do about it; the public learned there’s no point in calling about odor complaints.”

We read to interviewees a list of types of people who may have contacted their agency about public health concerns (Appendix S1). All participants said they had been contacted by individuals describing their own concerns, and most also said they had been contacted by members of an organized campaign regarding CAFOs/IFAP. Few agencies were contacted by health care providers.

We also questioned staff members about topics related to animal agriculture that their agencies may have ever been contacted about. A list of topics was provided, and all interviewees answered affirmatively regarding odor. Table 1 presents a ranking based on the number of interviewees reporting their agency had been contacted about the issue as it relates to animal agriculture. Rankings varied little between permitting and agriculture agency staff.

**Table 1:** Topics of concern ranked in descending order according to number of agency staff stating people have ever contacted the agency about the issue.

Odor
Respiratory health
Ground water quality/contaminated well
Violations of regulations
Waste getting on property
General health
Traffic

### Response to Citizen Concerns

Permitting agency staff reported a variety of responses depending on the concern; they often mentioned gathering additional information and checking the validity of a concern by contacting the person reporting the issue or by investigating the issue. Inspection activities were frequently delegated to regional field staff or county conservation district staff. Permitting agencies also made referrals to other agencies including departments of agriculture and health, as well as state Farm Bureaus (agriculture trade group), when issues were beyond their jurisdictions. Staff indicated that at times they would contact the person who had raised the concern to let them know that they could not address it. One interviewee said: “If the problem is not covered under the agency, it might be a phone call or email to let people know why we can’t address their concerns. Water issues are our primary jurisdiction. There are no state/federal regulations over air emissions.” The person was referring to a lack of air quality regulations in their state. As mentioned above, only two states reported that they were able to address some air quality concerns under their current regulatory authority. A staff member in a state with an air quality standard that applies to all IFAP operations (i.e., sites that existed when the regulation was adopted and new sites) noted that if there were a “major complaint” they would use their equipment to take air samples.

Some agency staff reported reaching out to producers directly to try to resolve issues. One interviewee described an agreement with the state Farm Bureau in which the agriculture group would send someone to talk to the producer “farmer to farmer” in the event of a reported concern. They thought that producers are more willing to speak to another farmer than to an agency staff member, and that this process results in issues being resolved faster. Another staff member described talking to producers initially to see if rules are being followed. Then they explain the concern and determine if it can be resolved without further agency action. Some concerns could be related to a regulatory violation, but few agency personnel described a process for enforcement in response to community member concerns. Based on interviewees’ responses, this phenomenon appeared to reflect factors including limited jurisdiction over common concerns and preference for voluntary and informal solutions to violations of regulations. All permitting agency interviewees stated that records were maintained of reported concerns, but it was not clear if those records always included concerns that were not formally investigated.

Agriculture department staff members’ responses to concerns varied due to the state-by-state differences in agriculture agencies’ jurisdiction over IFAP. In general, staff said that they investigate and/or refer to relevant agencies in response to concerns. Staff members from several states said they would refer concerns to the state permitting agency, either right away or after learning more about the situation. Most department of agriculture interviewees indicated that they had limited authority over many IFAP concerns and said the state permitting agencies would be more involved. Issues related to manure or dead animals, however, were more likely to be dealt with by agriculture departments. A small number of interviewees said they might contact the state health department in response to a call from a concerned citizen, although one said specifically that they would not. As with permitting agencies, several department of agriculture staff members said they try to work directly with producers to resolve issues. No interviewees in agriculture departments talked about enforcement or penalties that would be imposed by their department after investigating a reported concern. Most staff members said no records were kept of concerns; one said they kept records and another said records are kept only if they investigate the concern.

### Potential Role of Health Departments

The majority of permitting agency staff members and all agriculture department personnel interviewed thought that health departments should play a role in resolving citizen concerns related to IFAP. Interviewees indicated that they believed health departments should respond to health related concerns, work on air quality issues, provide technical assistance, collect and disseminate relevant data, and/or participate and provide input when regulations are under consideration. A small number of permitting agency and department of agriculture interviewees were not sure what the role of health departments should be, since health departments did not have regulatory mandates to enforce. Some staff stressed the importance of health department involvement due to permitting and agriculture agencies’ lack of health expertise. One permitting agency staff member stated, “If anyone’s going to [address health issues], it would have to be the health departments. From our perspective, we don’t really have expertise in that area,” and another said, “Obviously the health department has more expertise in the health area than us.” Some agriculture agency staff members thought that involving health departments would provide additional assurance to the public that departments of agriculture are unable to provide on health issues.

### Additional Engagement

#### Education

We asked interviewees if they perform any health education activities related to “animal production farms,” and the majority of permitting agency staff members said that they present information to producer/farmer groups in order to keep them informed on current regulations and requirements. A few talked about presenting to community groups when asked to do so, and that such presentations also addressed current regulatory requirements. Agriculture agency staff members also said that they provided information to farmers/producers and agriculture groups on current regulations, and at times presented information to community groups. No staff member, in permitting or agriculture agencies, said that they provided information regarding potential health issues related to IFAP.

#### Data collection

In response to a query about collection of environmental monitoring or health data, most permitting agency participants said that their agencies did little or no routine data collection. Data collection was usually performed in response to a concern, and generally involved surface water and well testing. One interviewee said, “If we have a well water complaint we’ll do sampling case by case. We need to be sure there is good evidence the well is tainted or everyone would want their well water tested at our expense.” One staff member said that some permits they issue require water monitoring and they do some monitoring at inspections, and that the data they collect is used for internal purposes only. One permitting staff member said they collected data on air quality in the past because they were directed to do so by the state legislature and that that monitoring led to a rulemaking process for farm emissions. The legislature, however, ultimately prevented the rule from being finalized and implemented. No agriculture department staff member reported that they collected environment or health data, but one interviewee said their department was working with a university to look into air quality issues.

#### Contact with community/environment groups

All permitting agency personnel reported some contact with organizations or groups of citizens that work to address local animal production farm issues. A few interviewees said their contact with community groups primarily consisted of receiving comments when new operations were proposed or receiving repeated requests for increased monitoring and stricter regulations. In one state, citizens petitioned the U.S. EPA to have the permitting agency’s delegated authority over CAFOs revoked because they believed the agency was not doing enough to regulate CAFOs in the state. At the time of the interview, the action was still pending and six new staff had been hired in response to the petition. Another interviewee said that environment groups serve as “watch dogs” and are “good partners from that standpoint” because they look for problems, conduct their own monitoring, and quickly contact the agency about problems so they can investigate. One staff member spoke about serving on an advisory committee with community representatives and another said a group working to revise their state’s manure manual, a guidance document for producers, includes citizen groups.

Most agriculture department staff said community/environment groups contact them, and some said that they have worked with them to address issues of concern. One interviewee said there are fewer groups now due to improved regulations in their state, and another said contacts from these groups increase in frequency when new operations are proposed.

### Barriers and Needs

While most interviewees appeared to consider their agency’s current level of engagement with IFAP to be appropriate, many staff members did describe a number of barriers preventing them from implementing their current oversight efforts more fully and from expanding their efforts to monitor IFAP operations and respond to concerns.

#### Barriers

Permitting agency staff described the main barriers affecting their oversight of IFAP as limited budgets, staff size, and political factors. Some also mentioned that recent budget and staff reductions due to the economic downturn have affected their departments’ ability to meet their regulatory obligations. Interviewees spoke about the effects of budget cuts on inspection frequency and farm visits, resources available for environmental monitoring, and ability of staff to conduct or attend in-person meetings and provide technical assistance. One staff member said “face to face meetings are much more effective when working with agriculture, and staff cutbacks are not good for that,” and another said that they “would like to spend more time working with producers so they know the regulations and what causes water problems.” Some interviewees said political factors have prevented them from more effectively regulating IFAP. For example, one permitting agency staff member explained that they had attempted to begin surface water monitoring at farms in response to concerns from environmental groups, but that financial and political issues had prevented this from moving forward. Only one agriculture agency staff member responded to an open-ended question regarding barriers with concerns about the agency’s budget, but when agriculture departments were presented with a list of potential needs (Table 2), all staff members responded affirmatively regarding the need for increased funding.

**Table 2:** Needs indicated by interviewees from list (Descending order).

Permitting Agency Staff
More staff dedicated to environmental health
Increased funding for environmental health
Educational materials for distribution
Funding specifically for animal production activities
Training for staff on public health issues relevant to animal production farms
Different political climate
Information on health effects of concern
Environmental quality tracking tools
Clearer federal regulations/guidelines (item added during study in response to data collected; not asked of all interviewees)
Connections to experts (i.e., university researchers)
**Agriculture Department Staff**
Increased funding
Updated information from researchers on health effects of concern
More staff
Funding specifically for animal production activities
Educational materials for distribution
Training for staff on issues relevant to animal production farms
Different political climate
Connections to experts
Environmental quality tracking tools

The threat of legal action was an additional difficulty and barrier brought up by some permitting agency staff members and one agriculture department representative. The agriculture department interviewee said they “feel uncomfortable responding when a group brings in a lawyer” because they are not prepared to respond to legal action against the agency. The permitting agency participants who mentioned lawsuits said specifically that federal regulations are unclear about who needs a permit, and that they worry about lawsuits stemming from the lack of clarity.

#### Needs

We asked staff an open-ended question about items that could increase their agencies’ effectiveness when addressing IFAP-related issues and further prompted them with a list of potential items (Appendix S1). The items most frequently identified as needs by interviewees are listed in Table 2. Some wording was adapted slightly in order to be more appropriate for and relevant to each agency. The most commonly expressed needs/resource improvements identified by permitting agency staff were: increased funding, more staff for environmental health, and educational materials for distribution to producers or the general public.

Even though most agriculture agency staff members did not mention funding as a barrier in an open-ended question about barriers/needs, it was the only item on the list that all agriculture interviewees said would be helpful to their department. Other needs identified were: updated information from researchers regarding the health effects of concern and more staff.

## Discussion

Our study reveals that sampled state permitting and agriculture agencies have taken limited actions to prevent and/or respond to public health concerns arising from IFAP operations. The main barriers identified that prevent further engagement include narrow or inadequate regulations, a lack of public health expertise within the agencies, and limited resources. There was widespread agreement among permitting and agriculture agency interviewees that health departments (HDs) should play a role in regulating IFAP operations, partly due to their own agencies’ limited mandates and available expertise in public health. Yet previously published findings show limited involvement by local and state HDs due to political barriers and a lack of jurisdiction, expertise, and resources [36].

These results indicate a fragmented system to protect public health where no agency has ownership of monitoring or addressing the impact of IFAP on people’s health. In short, HDs generally lack jurisdiction over IFAP operations [36] and permitting and agriculture agencies generally lack jurisdiction over and the capacity to address public health concerns. A growing divide between environmental and public health agencies was identified in the 1990′s as a trend that threatens public health protections [42]. Research has found that the main foci of environment agencies have shifted to permitting, enforcement, record keeping, and standard setting, and away from public health evaluations [43]. Our findings are consistent with these trends.

Ideally, a new role for HDs in responding to community concerns over IFAP operations would be defined by legislation aimed at remedying the lack of explicitly health-focused protections in current IFAP regulations. Unfortunately, this seems unlikely at present due to economic and political factors at the local, state, and federal levels [44]. In the absence of needed legislative and regulatory reforms, we suggest that partnerships between HDs and agriculture and permitting agencies could begin to improve the situation by including public health considerations in local and state-level decisions regarding the permitting, monitoring, and enforcement of regulations pertaining to IFAP operations. For example, greater agriculture and permitting agency collaboration with HDs could result in more comprehensive setback requirements for IFAP operations or the implementation of air quality standards for IFAP emissions linked to health concerns. Given the aforementioned support that several study participants voiced for greater involvement of HDs in IFAP regulation, agencies appear to be receptive to these partnerships. The drawback of this approach, as compared to new regulations, is that these relationships would have to be created one-by-one and maintained over time in the absence of regulations providing HDs with a defined role. We also suggest a potential approach whereby panels of experts on IFAP and public health could develop training programs and provide technical support to agencies interested in such partnerships in order to help foster their development and improve effectiveness. Future work should seek to examine evidence-based strategies that can inform and serve as models for these types of collaborations.

We did not seek to fully characterize how IFAP regulations are implemented by sampled agencies, but we are concerned that there is significant variability in inspection frequency among states. The inconsistency reflects varying requirements by state with no federal requirement for inspection frequency [31]. Further investigation is also needed of the practice of state agencies contacting farmer and agriculture trade groups for assistance when called about a concern. It is possible that this is an effective way to deal with minor issues; however, if problems that could result in fines and other regulatory action are routinely handled in an informal manner, the deterrent function of penalties for poor practices may be weakened and/or lost [45]. In addition, concerns about vulnerability to legal action stemming from a lack of clarity in federal regulations could cause agencies to be less aggressive in their enforcement of regulations. Additional research is needed to determine if this more informal approach to enforcement puts the environment and public health at greater risk than a formal approach to the enforcement of IFAP regulations.

### Strengths and Limitations

To our knowledge, this is the most in-depth study to date investigating how state agencies with jurisdiction over IFAP operations respond to health concerns. The value of the findings is amplified when combined with previously published results on the engagement of HDs with this issue. Including multiple agencies that could be involved with IFAP and public health–health, permitting, and agriculture agencies–allowed us to compile a more comprehensive profile of a system to protect public health that has substantial gaps. This approach was especially critical since previously published results showed that HDs routinely refer concerned citizens to permitting and agriculture agencies, with unknown outcomes for those referrals [36]. These findings may hold relevance for other issues characterized by problematic public health system gaps.

This study provides new information on a relatively unexplored topic; but the sample size was small, and the results cannot be interpreted as representing all agriculture and permitting agencies in the US. Also, we interviewed only one staff member per agency, and other staff members at the sampled agencies might have provided different responses to our questions. Finally, as with all studies of this nature, our sample reflects only those agencies that agreed to participate in the study.

## Conclusion

In light of steadily increasing evidence regarding the multifaceted impact of IFAP on the public’s health, it is crucial to examine how regulatory agencies respond to concerns and to understand what factors encourage and restrict agency staff from addressing health issues. A fragmented regulatory approach, narrow regulations, a lack of health expertise among agency staff, and limited resources are barriers preventing effective responses by sampled state permitting and agriculture agencies, despite the fact that jurisdiction over IFAP lies with these agencies. These findings are particularly troubling in light of prior research indicating that HDs also face multiple barriers to engagement with IFAP. The human health implications of IFAP operations have thus largely fallen by the wayside from a regulatory perspective, with rural communities suffering the consequences.

Given the near absence of explicit public health protections in current IFAP regulations, and the findings about responses from government agencies when issues are brought to their attention, there is a clear need for a more comprehensive public health response to IFAP. New regulations giving HDs a formal role in regulating IFAP and/or requiring public health experts on staff at regulatory agencies would go a long way toward addressing current gaps in the system. Short of new regulations, many actions could be taken to encourage capacity building and partnering among agencies. Future efforts should determine best practices for establishing these types of partnerships and evaluate their effectiveness in addressing the current disconnect between environmental regulations, public health, and IFAP. That said, voluntary efforts should not be seen as a replacement for much needed regulatory reforms. The public health implications of IFAP are increasingly clear, and regulations should ensure proper monitoring, oversight, and response by government agencies to protect public health.

## Supporting Information

Appendix S1 **Permitting Agency and Department of Agriculture Staff Member Questionnaires.**
(DOCX)
