# Supplementary material for: Investigating the Role of State Permitting and Agriculture Agencies in Addressing Public Health Concerns Related to Industrial Food Animal Production
Source: PLoS One. 2014 Feb 24;9(2):e89870. doi: 10.1371/journal.pone.0089870 (PMC3933695; doi:10.1371/journal.pone.0089870)
Supplement: Appendix S1 — Permitting Agency and Department of Agriculture Staff Member Questionnaires. (DOCX) [file pone.0089870.s001.docx]

Appendix S1: Permitting Agency and Department of Agriculture Staff Member Questionnaires

**1. Permitting Agency Staff Member Questionnaire**

**Confidentiality Statement**

Your personal information will be kept confidential. Your name and position will not be reported in the results of this study, and your responses will not be linked to your state. Your personal information and responses will be kept on password-protected computers and will not be shared outside of the research team.

1.  What is your position at the agency?

2. How many years have you been working at the agency?

3. Are permits for animal production farms handled at the county or state level?

4. What information is required for your agency to issue a NPDES permit or other permit for a new or expanding animal production farm?

5. Are there any restrictions on animal production farms in your state related to human health concerns (i.e. local zoning restrictions, setback requirements, moratorium on new or expanding sites, or health impact assessment requirement)?

6. Has your agency been contacted about concerns that people were associating with living or spending time near animal production farms or manure?

6a. If contacted, approximately how many times in a typical year? Has this changed over time and if so, how?

6b. Who made these contacts?

*(Do not supply list/examples to interviewee unless they ask for clarification.)*

1. Individual describing own concerns

2. Health care provider

3. Members of an organized campaign

4. Other (please describe)

6c. Are the contacts you receive about this issue spread out across the state, or are they mainly coming from certain regions?

7. What concerns were described?

*(Do not supply list/examples to interviewee unless they ask for clarification.)*

a. Bothersome odor

b. Respiratory health (i.e. asthma)

c. General health

d. Ground water quality/contaminated well

e. Waste getting on property

f. Violations of regulations

g. Traffic

h. Other (please describe)

8. Who (what position) within the agency is tasked with responding to such concerns?

9. Is there a set process in your department for responding to complaints about animal production farms, or are they handled on a case-by-case basis?

10. What is usually done in response to a complaint?

*If a referral to another Department is mentioned, ask who specifically they refer it to, and/or what section of the department.*

10a. So, just as an example, what would you do if someone called your department to complain about headaches associated with odor coming from an animal production facility?

11. Are records kept of complaints and responses?

12. Has your department carried out any education activities relevant to concerns about animal production farms?  If so, please describe.

13. Has your department collected any data relevant to animal production farms?  Information collected could include: private well testing, air/water monitoring, symptoms experiences by residents, etc.

13a. If so, what type of data was collected?

13b. Were the locations of animal production farms or manure spray fields taken into account when data was collected or analyzed?

13c. Has this information been written up in a report or distributed in some way?

14. Has your department worked with other local, state, or federal government agencies to respond to concerns about animal production farms?  If so, please describe.

15. Do you think health departments (county or state) should play a formal role in addressing health concerns relevant to animal production farms? Why or why not?

15a. If yes, what role(s) do you think would be appropriate for health departments?

16. Are there any organizations or groups of citizens in your county/state that work to address local animal production farm issues?  If so, has your agency ever worked with or been in contact with them? Please describe.

17. Please describe any additional actions your department has considered, but has not taken due to financial, political, capacity or other barriers.  (Please describe action that was considered and barrier.)

18. Does your agency need certain resources/circumstances to enable you to more effectively address concerns associated with having animal production farms in your state/county?  Please describe (select all that apply).

*(Leave question open-ended and then present list.)*

a. More staff

b. Increased funding

c. Funding specifically for animal production farm activities

d. Training for staff on issues relevant to animal production farms

e. Different political climate

f. Connections to experts

g. Updated information from researchers on env. & health effects of concern

h. Educational materials for distribution

i. Environmental quality tracking tools

j. Changes or clarifications in federal regulations

k. Other (please describe)

18a. *If they mention a different political climate AND other resources:*

If the political climate stayed the same as it is now, how would you make use of the other resources?

19. We are also contacting state departments of agriculture as part of this study. Is there anyone in particular who works on animal production farm issues that you suggest we contact in your state?

20. Is there anything else you would like to add that you did not have an opportunity to share?

***Thank you for participating!***

**2. Department of Agriculture Staff Member Questionnaire**

**Confidentiality Statement**

Your personal information will be kept confidential. Your name and position will not be reported in the results of this study, and your responses will not be linked to your state. Your personal information and responses will be kept on password-protected computers and will not be shared outside of the research team.

1. What is your position at the agency?

2. How many years have you been working at the agency?

3. Has your agency been contacted about concerns that people were associating with living or spending time near animal production farms or manure?

3a. If contacted, approximately how many times in a typical year? Has this changed over time and if so, how?

3b. Who made these contacts?

*(Do not supply list/examples to interviewee unless they ask for clarification.)*

1. Individual describing own concerns

2. Health care provider

3. Members of an organized group

4. Other (please describe)

3c. Are the contacts you receive about this issue spread out across the state, or are they mainly coming from certain regions?

4. What concerns were described?

*(Do not supply list/examples to interviewee unless they ask for clarification.)*

a. Bothersome odor

b. Respiratory health (i.e. asthma)

c. General health

d. Ground water quality/contaminated well

e. Waste getting on property

f. Violations of regulations

g. Traffic

h. Other (please describe)

5. Who (what position) within the agency is tasked with responding to such concerns?

6. Is there a set process in your department for responding to complaints about animal production farms, or are they handled on a case-by-case basis?

7. What is usually done in response to a complaint?

*If a referral to another Department is mentioned, ask who specifically they refer it to, and/or what section of the department.*

7a. So, just as an example, what would you do if someone called your department to complain about headaches associated with odor coming from an animal production facility?

8. Are records kept of complaints and responses?

9. Has your department carried out any education activities relevant to concerns about animal production farms? If so, please describe.

10. Has your department collected any data relevant to animal production farms? Information collected could include: private well testing, air/water monitoring, symptoms experiences by residents, etc.

10a. If so, what type of data was collected?

10b. Were the locations of animal production farms or manure spray fields taken into account when data was collected or analyzed?

10c. Has this information been written up in a report or distributed in some way?

11. Has your department worked with other local, state, or federal government agencies to respond to concerns about animal production farms? If so, please describe.

12. Do you think health departments (county or state) should play a formal role in addressing health concerns relevant to animal production farms? Why or why not?

12a. If yes, what role(s) do you think would be appropriate for health departments?

13. Are there any organizations or groups of citizens in your county/state that work to address local animal production farm issues? If so, has your agency ever worked with or been in contact with them? Please describe.

14. Please describe any other activities your department has performed to address issues relevant to animal production farms in your area.

15. Please describe any additional actions your department has considered, but has not taken due to financial, political, capacity or other barriers. (Please describe action that was considered and barrier.)

16. Does your agency need certain resources/circumstances to enable you to more effectively address concerns associated with having animal production farms in your state/county? Please describe (select all that apply).

*(Leave question open-ended and then present list.)*

a. More staff

b. Increased funding

c. Funding specifically for animal production farm activities

d. Training for staff on issues relevant to animal production farms

e. Different political climate

f. Connections to experts

g. Updated information from researchers on health effects of concern

h. Educational materials for distribution

i. Environmental quality tracking tools

j. Other (please describe)

16a.  *If they mention a different political climate AND other resources:*

If the political climate stayed the same as it is now, how would you make use of the other resources?

17. Is there anything else you would like to add that you did not have an opportunity to share?

***Thank you for participating!***
